# Catecholamines Mediate Multiple Fetal Adaptations during Placental Insufficiency That Contribute to Intrauterine Growth Restriction: Lessons from Hyperthermic Sheep

**DOI:** 10.1155/2011/740408

**Published:** 2011-05-11

**Authors:** D. T. Yates, A. S. Green, S. W. Limesand

**Affiliations:** Department of Animal Sciences, The University of Arizona, 1650 E. Limberlost Drive, Tucson, AZ 85719, USA

## Abstract

Placental insufficiency (PI) prevents adequate delivery of nutrients to the developing fetus and creates a chronic state of hypoxemia and hypoglycemia. In response, the malnourished fetus develops a series of stress hormone-mediated metabolic adaptations to preserve glucose for vital tissues at the expense of somatic growth. Catecholamines suppress insulin secretion to promote glucose sparing for insulin-independent tissues (brain, nerves) over insulin-dependent tissues (skeletal muscle, liver, and adipose). Likewise, premature induction of hepatic gluconeogenesis helps maintain fetal glucose and appears to be stimulated by both norepinephrine and glucagon. Reduced glucose oxidation rate in PI fetuses creates a surplus of glycolysis-derived lactate that serves as substrate for hepatic gluconeogenesis. These adrenergically influenced adaptive responses promote *in utero* survival but also cause asymmetric intrauterine growth restriction and small-for-gestational-age infants that are at greater risk for serious metabolic disorders throughout postnatal life, including obesity and type II diabetes.

## 1. Introduction

Placental insufficiency (PI) is a common cause of fetal malnutrition that manifests into small-for-gestational-age (SGA) infants [1]. Poor nutrient supply necessitates metabolic adaptations by the fetus that result in asymmetrical intrauterine growth restriction (IUGR) [2–4]. Although specific etiology of PI is rarely determined and is likely case-specific, common results of placental dysfunction include fetal hypoxemia and hypoglycemia that worsen in concert with increasing fetal demands over the last half of gestation, causing hypercatecholaminemia and hypoinsulinemia [4–7]. Parallels for characteristics observed in human PI-IUGR fetuses have been demonstrated in an ovine model of hyperthermia-induced PI [8]. Similar to humans, body mass in ovine PI-IUGR fetuses is normal at the end of the first trimester but is reduced by 25% at the end of the second trimester [9, 10] and by over 50% near term [11]. Restricted, asymmetrical growth is a consequence of adaptive responses that support critical organ development at the expense of somatic growth [10]. Epidemiological findings have linked PI-induced alteration of fetal metabolic phenotype to greater incidence of postnatal metabolic complications such as hypertension [12], obesity [13], and type II diabetes [14–16]. The hyperthermia-induced ovine model of PI-IUGR provides an *in vivo* system for conducting comparative physiological studies of fetal developmental adaptations during pathophysiology [8]. This paper highlights findings regarding fetal adaptive responses learned from this ovine model of PI-IUGR.

## 2. Experimental Placental Insufficiency: The Hyperthermic Ovine Model

Hyperthermically induced PI-IUGR has been compared to other forms of IUGR induction in sheep, and advantages of this model have been described [8, 17]. To induce PI, pregnant ewes are placed in environmentally controlled chambers beginning at the 40th day of gestation (dGA; term is 147 dGA) and exposed to elevated ambient temperatures in a diurnal pattern of 40°C for 12 hours and 35°C for 12 hours (dew point 22°C; relative humidity 35–45%) for 55 consecutive days, though severity of growth restriction may be reduced by decreasing duration of exposure [18]. In concert with high ambient temperatures, ewes exhibit a 0.7–1.0°C rise in core body temperature [19, 20]. This febrile response is a hypothesized source of vasopressin-mediated reduction in maternal blood flow to the uterus [19], which slows placental growth and development. A smaller, less metabolically active placenta benefits the hyperthermically stressed dam by producing less heat and requiring fewer nutrients, but the trade-off of stunted growth and development is a placenta that cannot adequately meet nutritional demands of the growing fetus in later stages of gestation [21]. 

Ewes exposed to hyperthermic conditions described above exhibit slightly lighter placentas than pair-fed thermoneutral contemporaries beginning at approximately 75 dGA (~0.5 gestation), but the disparity between normal and compromised placentas worsens with increasing gestational age and mass may differ by as much as 66% at term [18, 22]. Reduced placental mass results from less total tissue and smaller, rather than fewer placentomes [18, 22, 23]. Additionally, cotyledons, the fetal tissue in placentomes, exhibit poor vascular organization [24]. In normal pregnancies, the need for oxygen within the growing placenta stimulates release of vascular endothelial growth factor (VEGF) to promote branching angiogenesis [25]. Angiogenic growth factors are responsible for the 10-fold increase in capillary area density and 14-fold increase in total villous surface area that occurs between early and late gestation [26]. However, when oxygen content within the early stage placenta becomes abnormally low due to reduced uterine blood flow, a premature spike in VEGF disrupts the intended progression of vasculargenesis [27] and may result in a period of placental hyperoxia [10, 28, 29]. In this hyperoxic state, the incentive to secrete angiogenic growth factors is lost, resulting in underdevelopment of placental vasculature structure [29].

## 3. Placental Insufficiency Causes Fetal Malnutrition

**Figure 1:**
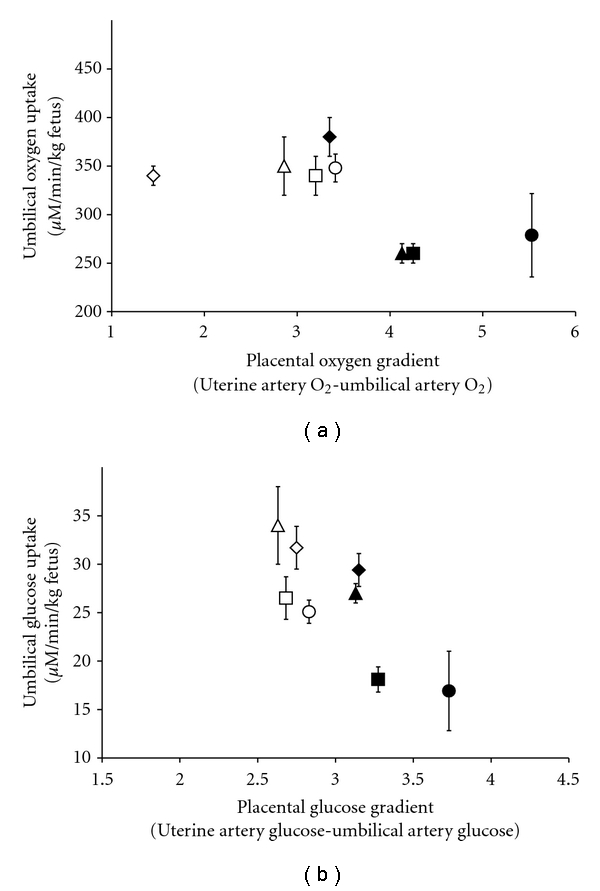
Increased transplacental oxygen and glucose gradients appear to partially ameliorate rates of umbilical uptake in hyperthermia-induced PI-IUGR fetuses (black symbols) compared to uncompromised fetuses (white symbols). Data are from Thureen et al. [23] (diamonds), Limesand et al. [30] (squares) and [31] (circles), and De Vrijer et al. [32] (triangles).

Increased oxygen and glucose gradients between maternal and fetal circulation are hallmarks of PI, and appear to help promote transplacental diffusion [23, 30–32] (Figure 1). Nevertheless, umbilical oxygen content is diminished by as much as 50% following hyperthermically induced PI at 0.9 of gestation [31], despite normal oxygen concentrations in uterine blood. Oxygen exchange between maternal and fetal circulation occurs by simple diffusion and without assistance from specialized transporters. Thus, oxygen transport rate across the placenta is determined by perfusion rate, placental surface area, and distance between maternal and fetal vasculature. Smaller placental mass and vascular disorganization contribute to reduced oxygen exchange but may be partially offset by reduced thickness of tissue and increased transplacental gradient so that absolute umbilical oxygen uptake and content are reduced after hyperthermic insult even though umbilical oxygen uptake *per unit of placental mass* is similar between hyperthermic and thermoneutral animals [23, 31, 33, 34].

**Figure 2:**
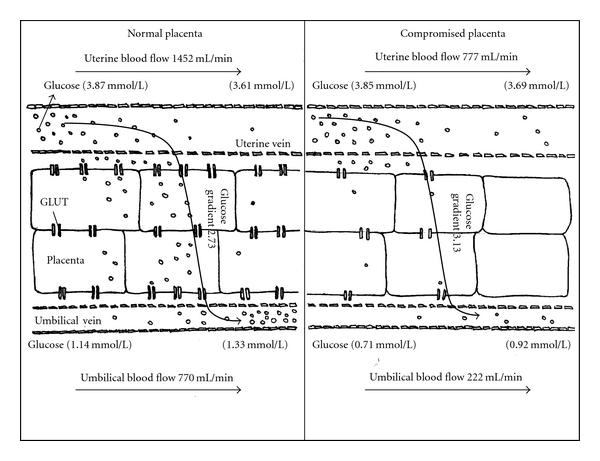
A schematic representation of glucose transfer across normal and PI-IUGR placenta. Facilitated diffusion of glucose occurs down its concentration gradient through membrane-bound GLUT transporters in normal and compromised placentas. Values are from Regnault et al. [11].

Umbilical vein glucose concentrations are also approximately 50% lower in PI-IUGR fetuses during late gestation [23, 33]. Unlike placental oxygen transport, placental glucose exchange requires facilitative glucose transporters for transfer of glucose down its maternal-to-fetal concentration gradient (Figure 2). Although less placental mass and surface area contributes to reduced absolute glucose transport, placental glucose transport *per unit of placental mass* is also lower. The later observation is associated with reduced expression of glucose transporter transcripts 1, 3, and 8 [30, 33], which were previously identified in ovine placental tissue [30, 35, 36]. 

**Figure 3:**
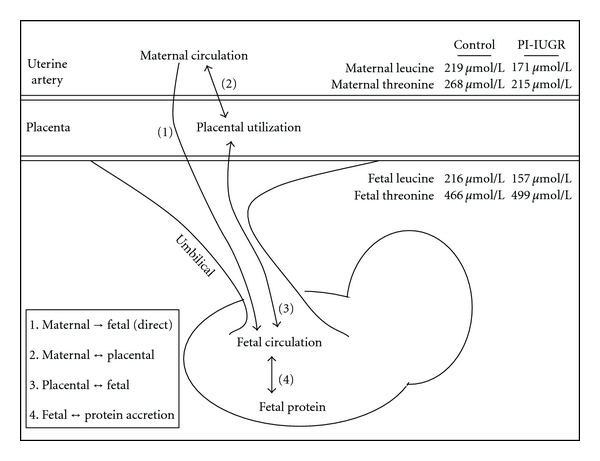
Amino acid fluxes between maternal circulation, placental tissue, and fetal circulation. Values are from Ross et al. [38] and Anderson et al. [37].

Umbilical uptake of amino acid is also diminished (23% and 58% in absolute uptake and uptake *per unit of placental mass*, resp.) [32, 37, 38], likely due to reduced placental mass, surface area, and transporter expression (Figure 3). Because amino acids are actively transported and may be utilized or otherwise altered by the placenta, flux occurs in several directions simultaneously: maternal → fetal circulation (direct), maternal *↔* placenta, and placenta *↔* fetal [39]. The rate of each flux is amino acid specific, and severity of reduced transport depends largely on which of several transport systems is the primary facilitator of that amino acid. Though not yet defined in the ovine PI-IUGR, Systems A, y+, and X^−^_AG_ are most profoundly affected in rat models [40, 41].

Fetal nutrient and oxygen requirements increase with growth and eventually surpass the capacity at which the compromised placenta can deliver oxygen and glucose to the fetus, creating a chronic state of hypoxemia and hypoglycemia that progressively worsens over the last half of gestation [11, 42]. PI-induced malnutrition elicits adaptive responses by the fetus that alter its physiological profile to support the most vital tissues at the expense of normal growth and development [43, 44]. 

## 4. Fetal Malnutrition Causes Hormone-Mediated Adaptive Responses

Fetal adaptations to PI-induced hypoxemia and hypoglycemia are mediated by endocrine responses (Table 1). Secretion of the catecholamines, norepinephrine and epinephrine, from the adrenal medulla, increases drastically in response to hypoxemia [45]. In addition to hypothalamic stimulation via the splanchnic nerve during later gestation, low oxemic levels stimulate chromaffin cells of the medulla directly via inactivation of oxygen-sensitive potassium channels [46]. The change in potassium concentration favors membrane depolarization needed for catecholamine exocytosis. Both norepinephrine [47] and epinephrine [48] are secreted in response to moderate fetal hypoxemia (12–18 mmHg), but epinephrine secretion is increased in greater proportion [49, 50], especially after splanchnic innervation develops [51]. Catecholamines appear to be primary mediating factors in maintaining glucose supply in the PI-IUGR by suppressing insulin secretion and altering the fetal metabolic phenotype (discussed in detail below). However, adenosine concentration within the interstitial fluid of neural and skeletal muscle tissue also increases during fetal hypoxemia and, although not traditionally considered a hormone, has been shown to elicit many of the same responses as catecholamines [52, 53]. Also, hypoglycemia-stimulated cortisol secretion from the adrenal cortex has been observed in the ovine fetus [54, 55], though stimulation during hyperthermia-induced PI-IUGR is debatable.

**Figure 4:**
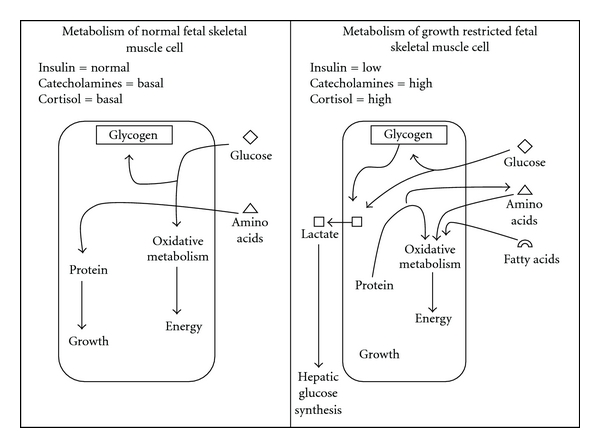
Proposed changes in metabolic phenotype of skeletal muscle in growth-restricted fetuses with placental insufficiency. Changes in the profiles of metabolic hormones likely cause a shift from glucose to amino acids/fatty acids as the primary substrates for oxidative metabolism. Intracellular glucose and glycogen are instead converted to lactate, which can then be transferred out of the cell and shuttled to the liver and resynthesized into glucose.

By binding and activating *α*2-adrenergic receptors on pancreatic *β* cells, catecholamines suppress insulin secretion and may reduce plasma insulin concentrations by ~5-fold in PI-IUGR [42] while glucagon secretion from pancreatic *α*-cells increases [56]. During chronic hypoinsulinemia, neural tissues, which are insulin independent, have a competitive advantage over tissues that rely on insulin-mediated uptake, such as liver, skeletal muscle, and adipose tissue. Moreover, GLUT1 transporters are upregulated in the brain to facilitate crucial glucose uptake at the expense of systemic growth [31, 69, 70]. Skeletal muscle cells enhance sensitivity to chronically low insulin by increasing insulin receptor concentrations, which restores glucose uptake [56, 71]. However, since glucose oxidation rates are depressed in the PI-IUGR fetus [31], enhanced insulin sensitivity in skeletal muscle likely restores glucose uptake for anaerobic metabolism and not oxidative energy production. Muscle cells appear to cope with catecholamine-mediated restriction of glucose oxidation through several mechanisms (Figure 4). Increased systemic oxidation rate of amino acids has been demonstrated in both PI-IUGR and experimentally hypoglycemic fetuses [37, 38, 72], indicating that muscle cells are relying on alternative substrates for oxidative energy production. Utilization of free fatty acids for energy is thought to be minimal in the uncompromised ovine fetus [73], but elevated catecholamines increase mobilization and *β*-oxidation in infantile lambs [57] and may promote similar use of fat in the late-term PI-IUGR fetus, though utilization by skeletal muscle has yet to be examined in this model. Consumption of amino acids for energy production coupled with reduced net influx from maternal circulation restricts protein accretion and muscular growth in the PI-IUGR fetus.

**Figure 5:**
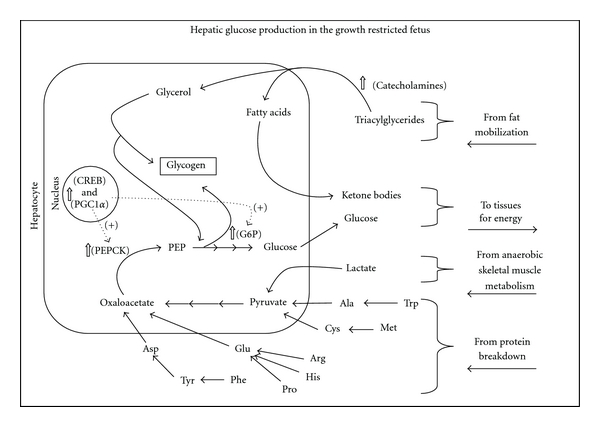
Glucose production by the liver occurs in placental insufficiency-induced growth-restricted fetuses to maintain glucose supply to vital tissues. Stress hormones initiate transcriptional cofactors responsible for expression of gluconeogenic enzymes. Lactate from anaerobic metabolism and amino acids from protein breakdown are gluconeogenic substrates, while fatty acids from adipose breakdown are converted to ketone bodies, which provide energy to neural tissue.

The metabolic end product of anaerobic glucose metabolism, lactate, can exit muscle cells into circulation. In addition to being observed in PI-IUGR fetuses [31], elevated plasma lactate concentrations have also been observed during acute catecholamine and adenosine infusion into uncompromised ovine fetuses [52, 74], indicating a regulatory role for these compounds. During PI, lactate becomes a crucial substrate for glycogen synthesis in cardiac tissue [75] and, in a process known as the Cori cycle, serves as a precursor for hepatic gluconeogenesis, along with amino acids and other substrates (Figure 5). In normal, well-nourished fetuses, gluconeogenesis does not develop until the final days of gestation in anticipation of postnatal life [76–78]. In the PI-IUGR, however, stress hormones appear to encourage much earlier establishment of hepatic glucose production by upregulating two major gluconeogenic enzymes, glucose-6-phosphatase (G6P) and phosphoenolpyruvate carboxykinase (PEPCK) [31, 78, 79], in part through increased levels of relevant transcriptional factors and coactivators [31, 54, 79]. Hepatic gluconeogenesis is crucial to maintaining the fetal glucose pool and likely preserves glycogen stores in muscle and liver tissues [31]. A final catecholamine-mediated energy source for neural tissue in the PI-IUGR fetus appears to be provided by conversion of mobilized fatty acids to ketone bodies by the liver, although this has not been demonstrated in the hyperthermic ovine model.

**Figure 6:**
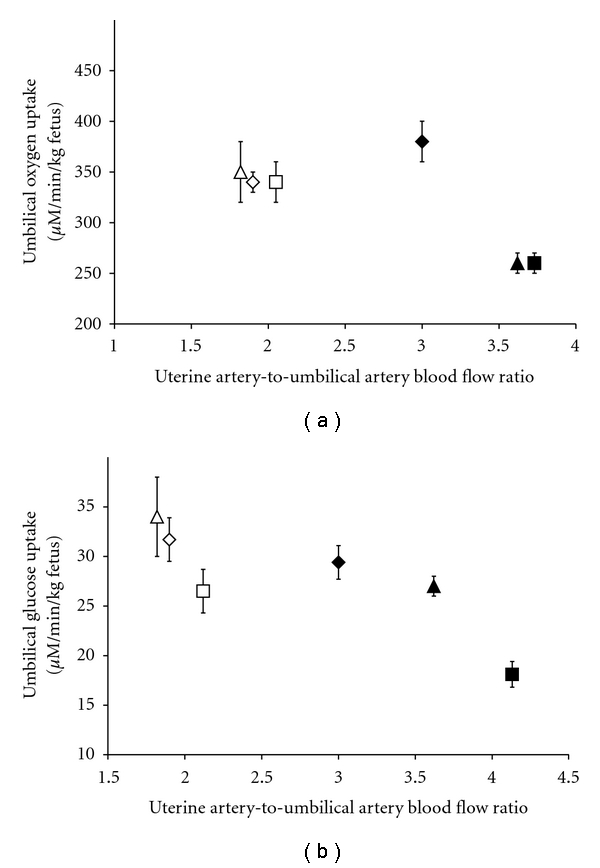
Increased uterine-to-umbilical blood flow ratios are needed to partially recover umbilical uptake rates in hyperthermia-induced PI-IUGR fetuses (black symbols) compared to uncompromised fetuses (white symbols). Data are from Thureen et al. [23] (diamonds), Limesand et al. [30] (squares) and [31] (circles), and De Vrijer et al. [32] (triangles).

In addition to metabolic changes, fetal blood flow is altered in PI-IUGR fetuses. Near term, blood flow to the brain and heart of hyperthermic fetuses is increased by 50% and 8%, respectively, at the expense of flow to the liver, lungs, small intestine, pancreas, and adipose tissue, which decreases noticeably [80, 81]. Additionally, slower rate of umbilical blood flow caused by increased resistance [82] increases transplacental oxygen and glucose gradients, aiding extraction from maternal circulation [32, 34, 83, 84] (Figure 6). 

## 5. Adaptive Responses Cause Postnatal Consequences

Fetal adaptive responses to PI cause SGA offspring to be at greater risk for postnatal metabolic complications due in large part to altered sensitivities of tissues to stress hormones and other stimulants. Chronically elevated fetal catecholamines eventually desensitize adipose tissue by downregulation of *β*2 adrenergic receptors, which impairs fat mobilization and promotes adiposity in PI-IUGR lambs [57]. Similarly, chronic adrenergic suppression of pancreatic *β*-cells causes compensatory adaptations that result in hypersensitivity to glucose stimulation and appear to cause oversecretion of insulin in the neonatal lamb (Limesand, unpublished data). This, coupled with enhanced sensitivity to insulin in skeletal muscle due to chronic hypoinsulinemia [56, 71], may increase propensity for glucose to be stored as fat during “catch-up” growth and predispose offspring to childhood obesity. Fetal adaptive responses are also reflected in aberrant anatomy of the pancreas. Glucagon-producing pancreatic *α*-cells exhibit normal responsiveness and morphometrics [56], but *β*-cells may be reduced in mass by as much as 75% in the PI-IUGR due to lower replication rate [85]. Correspondingly, fetal islets contain 80% less insulin content and are less able to induce glucose oxidative metabolism [31, 56]. Thus, the ability to synthesize and store insulin in pancreatic islets is reduced, though the ability to secrete what insulin is available in response to stimulation is actually greater, as described above. To illustrate, basal plasma insulin in human SGA infants was shown to be reduced at 48 hours of age, similar at 1 year of age, and greater at 3 years of age compared to infants of normal birth weight [58]. Moreover, glucose-stimulated insulin release was greater in these SGA infants at 1 and 3 years of age. Oversecretion of insulin in response to glucose stimulation may promote neonatal catch-up growth in SGA lambs [57, 86] and human infants [59, 87] that is based largely on fat deposition.

## 6. Summary

The ovine model of hyperthermia-induced PI-IUGR provides a comparative system for physiological and developmental research in the fetus that is applicable to human medicine. This model has been effectively used to study fetal adaptive responses to chronic oxygen and nutrient deprivation during placental insufficiency. Fetal responses appear primarily mediated by elevated concentrations of stress hormones, especially catecholamines, which seemingly influence changes in blood flow, endocrine profile, and cellular metabolism to support *in utero* survival. However, permanent fetal adaptations predispose offspring to obesity, type II diabetes, hypertension, and other serious metabolic complications throughout postnatal life.

## Figures and Tables

**Table 1:** Hormone profiles in ovine and human PI-IUGR fetuses and neonates.^1^

Hormone	Ovine				Human			
	Prenatal		Neonatal^2^		Prenatal		Neonatal^3^	
	Control	IUGR	Control	IUGR	Control	IUGR	Control	IUGR
Norepinephrine, pg/mL	636	2564	1073	?	132	1031	6500^4^	7400^4^
Epinephrine, pg/mL	Below detection	<40	394	?	Below detection	<180	360^4^	380^4^
Cortisol, ng/mL	4.4	4.4	28	19	1.66	1.13	1.24	0.96
Basal insulin, ng/mL	0.28	0.09	0.74	0.73	0.98	0.54	0.48	0.48
GSIS insulin, ng/mL	0.60	0.14	4.16	8.05	4.91	0.58	2.15	3.83
Glucagon, pg/mL	39.8	79.0	139	?	133	259	87	78

^1^Data from [4, 6, 56–68] (S. W. Limesand, unpublished).

^2^Preruminal lambs, 8–21 days of age.

^3^Infants, <1 yr of age.

^4^Measured in urine samples, normalized to creatinine concentration.

## References

[1] Creasy RK, Resnik R, Iams J (2004). *Maternal-Fetal Medicine: Principles and Practice*.

[2] Platz E, Newman R (2008). Diagnosis of IUGR: traditional Biometry. *Seminars in Perinatology*.

[3] Economides DL, Nicolaides KH, Gahl WA, Bernardini I, Evans MI (1989). Plasma amino acids in appropriate- and small-for-gestational-age fetuses. *American Journal of Obstetrics and Gynecology*.

[4] Setia S, Sridhar MG, Bhat V, Chaturvedula L, Vinayagamoorti R, John M (2006). Insulin sensitivity and insulin secretion at birth in intrauterine growth retarded infants. *Pathology*.

[5] Economides DL, Proudler A, Nicolaides KH (1989). Plasma insulin in appropriate- and small-for-gestational-age fetuses. *American Journal of Obstetrics and Gynecology*.

[6] Greenough A, Nicolaides KH, Lagercrantz H (1990). Human fetal sympathoadrenal responsiveness. *Early Human Development*.

[7] Okamura K, Watanabe T, Tanigawara S (1990). Catecholamine levels and their correlation to blood gases in umbilical venous blood obtained by cordocentesis. *Fetal Diagnosis and Therapy*.

[8] Barry JS, Rozance PJ, Anthony RV (2008). An animal model of placental insufficiency-induced intrauterine growth restriction. *Seminars in Perinatology*.

[9] De Vrijer B, Davidsen ML, Wilkening RB, Anthony RV, Regnault TRH (2006). Altered placental and fetal expression of IGFS and IGF-binding proteins associated with intrauterine growth restriction in fetal sheep during early and mid-pregnancy. *Pediatric Research*.

[10] Regnault TRH, Galan HL, Parker TA, Anthony RV (2002). Placental development in normal and compromised pregnancies—a review. *Placenta*.

[11] Regnault TRH, de Vrijer B, Galan HL, Wilkening RB, Battaglia FC, Meschia G (2007). Development and mechanisms of fetal hypoxia in severe fetal growth restriction. *Placenta*.

[12] Vickers MH, Breier BH, Cutfield WS, Hofman PL, Gluckman PD (2000). Fetal origins of hyperphagia, obesity, and hypertension and postnatal amplification by hypercaloric nutrition. *American Journal of Physiology*.

[13] Desai M, Gayle D, Babu J, Ross MG (2005). Programmed obesity in intrauterine growth-restricted newborns: modulation by newborn nutrition. *American Journal of Physiology*.

[14] Barker DJP, Hales CN, Fall CHD, Osmond C, Phipps K, Clark PMS (1993). Type 2 (non-insulin-dependent) diabetes mellitus, hypertension and hyperlipidaemia (syndrome X): relation to reduced fetal growth. *Diabetologia*.

[15] Ravelli ACJ, Van Der Meulen JHP, Michels RPJ (1998). Glucose tolerance in adults after prenatal exposure to famine. *Lancet*.

[16] Newsome CA, Shiell AW, Fall CHD, Phillips DIW, Shier R, Law CM (2003). Is birth weight related to later glucose and insulin metabolism?—a systematic review. *Diabetic Medicine*.

[17] Green AS, Rozance PJ, Limesand SW (2010). Consequences of a compromised intrauterine environment on islet function. *Journal of Endocrinology*.

[18] Galan HL, Hussey MJ, Barbera A (1999). Relationship of fetal growth to duration of heat stress in an ovine model of placental insufficiency. *American Journal of Obstetrics and Gynecology*.

[19] Dreiling CE, Carman 3rd. F.S. FS, Brown DE (1991). Maternal endocrine and fetal metabolic responses to heat stress. *Journal of Dairy Science*.

[20] Regnault TRH, Orbus RJ, Battaglia FC, Wilkening RB, Anthony RV (1999). Altered arterial concentrations of placental hormones during maximal placental growth in a model of placental insufficiency. *Journal of Endocrinology*.

[21] Wells JCK (2002). Thermal environment and human birth weight. *Journal of Theoretical Biology*.

[22] Alexander G, Williams D (1971). Heat stress and development of conceptus in domestic sheep. *Journal of Agricultural Science*.

[23] Thureen PJ, Trembler KA, Meschia G, Makowski EL, Wilkening RB (1992). Placental glucose transport in heat-induced fetal growth retardation. *American Journal of Physiology*.

[24] Regnault TRH, Orbus RJ, De Vrijer B (2002). Placental expression of VEGF, PlGF and their receptors in a model of placental insufficiency—intrauterine growth restriction (PI-IUGR). *Placenta*.

[25] Arroyo JA, Winn VD (2008). Vasculogenesis and angiogenesis in the IUGR placenta. *Seminars in Perinatology*.

[26] Reynolds LP, Borowicz PP, Vonnahme KA (2005). Animal models of placental angiogenesis. *Placenta*.

[27] Lyall F, Young A, Boswell F, Kingdom JCP, Greer IA (1997). Placental expression of vascular endothelial growth factor in placentae from pregnancies complicated by pre-eclampsia and intrauterine growth restriction does not support placental hypoxia at delivery. *Placenta*.

[28] Kingdom J, Huppertz B, Seaward G, Kaufmann P (2000). Development of the placental villous tree and its consequences for fetal growth. *European Journal of Obstetrics Gynecology and Reproductive Biology*.

[29] Kingdom JCP, Kaufmann P (1997). Oxygen and placental villous development: origins of fetal hypoxia. *Placenta*.

[30] Limesand SW, Regnault TRH, Hay WW (2004). Characterization of glucose transporter 8 (GLUT8) in the ovine placenta of normal and growth restricted fetuses. *Placenta*.

[31] Limesand SW, Rozance PJ, Smith D, Hay WW (2007). Increased insulin sensitivity and maintenance of glucose utilization rates in fetal sheep with placental insufficiency and intrauterine growth restriction. *American Journal of Physiology*.

[32] De Vrijer B, Regnault TRH, Wilkening RB, Meschia G, Battaglia FC (2004). Placental uptake and transport of ACP, a neutral nonmetabolizable amino acid, in an ovine model of fetal growth restriction. *American Journal of Physiology*.

[33] Wallace JM, Regnault TRH, Limesand SW, Hay WW, Anthony RV (2005). Investigating the causes of low birth weight in contrasting ovine paradigms. *Journal of Physiology*.

[34] Bell AW, Wilkening RB, Meschia G (1987). Some aspects of placental function in chronically heat-stressed ewes. *Journal of Developmental Physiology*.

[35] Ehrhardt RA, Bell AW (1997). Developmental increases in glucose transporter concentration in the sheep placenta. *American Journal of Physiology*.

[36] Wooding FBP, Fowden AL, Bell AW, Ehrhardt RA, Limesand SW, Hay WW (2005). Localisation of glucose transport in the ruminant placenta: implications for sequential use of transporter isoforms. *Placenta*.

[37] Anderson AH, Fennessey PV, Meschia G, Wilkening RB, Battaglia FC (1997). Placental transport of threonine and its utilization in the normal and growth-restricted fetus. *American Journal of Physiology*.

[38] Ross JC, Fennessey PV, Wilkening RB, Battaglia FC, Meschia G (1996). Placental transport and fetal utilization of leucine in a model of fetal growth retardation. *American Journal of Physiology*.

[39] Regnault TRH, De Vrijer B, Battaglia FC (2002). Transport and metabolism of amino acids in placenta. *Endocrine*.

[40] Malandro MS, Beveridge MJ, Novak DA, Kilberg MS (1996). Rat placental amino acid transport after protein-deprivation-induced intrauterine growth retardation. *Biochemical Society Transactions*.

[41] Rosso P (1977). Maternal-fetal exchange during protein malnutrition in the rat. Placental transfer of glucose and a nonmetabolizable glucose analog. *Journal of Nutrition*.

[42] Leos RA, Anderson MJ, Chen X, Pugmire J, Anderson KA, Limesand SW (2010). Chronic exposure to elevated norepinephrine suppresses insulin secretion in fetal sheep with placental insufficiency and intrauterine growth restriction. *American Journal of Physiology*.

[43] Hales CN, Barker DJP (1992). Type 2 (non-insulin-dependent) diabetes mellitus: the thrifty phenotype hypothesis. *Diabetologia*.

[44] Hales CN, Barker DJP (2001). The thrifty phenotype hypothesis. *British Medical Bulletin*.

[45] Jellyman JK, Gardner DS, Edwards CMB, Fowden AL, Giussani DA (2005). Fetal cardiovascular, metabolic and endocrine responses to acute hypoxaemia during and following maternal treatment with dexamethasone in sheep. *Journal of Physiology*.

[46] Adams MB, McMillen IC (2000). Actions of hypoxia on catecholamine synthetic enzyme mRNA expression before and after development of adrenal innervation in the sheep fetus. *Journal of Physiology*.

[47] Comline RS, Silver M (1966). Development of activity in the adrenal medulla of the foetus and new-born animal. *British Medical Bulletin*.

[48] Jones CT, Robinson RO (1975). Plasma catecholamines in foetal and adult sheep. *Journal of Physiology*.

[49] Cheung CY (1990). Fetal adrenal medulla catecholamine response to hypoxia-direct and neural components. *American Journal of Physiology*.

[50] Cohen WR, Piasecki GJ, Cohn HE, Susa JB, Jackson BT (1991). Sympathoadrenal responses during hypoglycemia, hyperinsulinemia, and hypoxemia in the ovine fetus. *American Journal of Physiology*.

[51] Comline RS, Silver M (1961). The release of adrenaline and noradrenaline from the adrenal glands of the foetal sheep. *The Journal of Physiology*.

[52] Maeda T, Koos BJ (2009). Adenosine A1 and A2a receptors modulate insulinemia, glycemia, and lactatemia in fetal sheep. *American Journal of Physiology*.

[53] Koos BJ, Maeda T (2001). Adenosine A2A receptors mediate cardiovascular responses to hypoxia in fetal sheep. *American Journal of Physiology*.

[54] Rozance PJ, Limesand SW, Barry JS (2008). Chronic late-gestation hypoglycemia upregulates hepatic PEPCK associated with increased PGC1*α* mRNA and phosphorylated CREB in fetal sheep. *American Journal of Physiology*.

[55] Limesand SW, Hay WW (2003). Adaptation of ovine fetal pancreatic insulin secretion to chronic hypoglycaemia and euglycaemic correction. *Journal of Physiology*.

[56] Limesand SW, Rozance PJ, Zerbe GO, Hutton JC, Hay WW (2006). Attenuated insulin release and storage in fetal sheep pancreatic islets with intrauterine growth restriction. *Endocrinology*.

[57] Chen X, Fahy AL, Green AS, Anderson MJ, Rhoads RP, Limesand SW (2010). *β*2-adrenergic receptor desensitization in perirenal adipose tissue in fetuses and lambs with placental insufficiency-induced intrauterine growth restriction. *Journal of Physiology*.

[58] Mericq V, Ong KK, Bazaes R (2005). Longitudinal changes in insulin sensitivity and secretion from birth to age three years in small- and appropriate-for-gestational-age children. *Diabetologia*.

[59] Soto N, Bazaes RA, Peña V (2003). Insulin sensitivity and secretion are related to catch-up growth in small-for-gestational-age infants at age 1 year: results from a prospective cohort. *Journal of Clinical Endocrinology and Metabolism*.

[60] Johansson S, Norman M, Legnevall L, Dalmaz Y, Lagercrantz H, Vanpée M (2007). Increased catecholamines and heart rate in children with low birth weight: perinatal contributions to sympathoadrenal overactivity. *Journal of Internal Medicine*.

[61] van Kempen AAMW, Ackermans MT, Endert E, Kok JH, Sauerwein HP (2005). Glucose production in response to glucagon is comparable in preterm AGA and SGA infants. *Clinical Nutrition*.

[62] Strinic T, Roje D, Marusic J, Capkun V (2007). Cord blood cortisol level is lower in growth-restricted newborns. *Journal of Obstetrics and Gynaecology Research*.

[63] Giudice LC, De Zegher F, Gargosky SE (1995). Insulin-like growth factors and their binding proteins in the term and preterm human fetus and neonate with normal and extremes of intrauterine growth. *Journal of Clinical Endocrinology and Metabolism*.

[64] Jefferies CA, Hofman PL, Keelan JA, Robinson EA, Cutfield WS (2004). Insulin resistance is not due to persistently elevated serum tumor necrosis-*α* levels in small for gestational age, premature, or twin children. *Pediatric Diabetes*.

[65] Hubinont C, Nicolini U, Fisk NM, Tannirandorn Y, Rodeck CH (1991). Endocrine pancreatic function in growth-retarded fetuses. *Obstetrics and Gynecology*.

[66] Chappell BA, Padbury JF, Habib DM (1991). Pulmonary clearance of norepinephrine in lambs. *Pediatric Research*.

[67] Louey S, Cock ML, Stevenson KM, Harding R (2000). Placental insufficiency and fetal growth restriction lead to postnatal hypotension and altered postnatal growth in sheep. *Pediatric Research*.

[68] Wallin LA, Fawcett CP, Rosenfeld CR (1989). Oxytocin stimulates glucagon and insulin secretion in fetal and neonatal sheep. *Endocrinology*.

[69] Sadiq HF, Das UG, Tracy TF, Devaskar SU (1999). Intra-uterine growth restriction differentially regulates perinatal brain and skeletal muscle glucose transporters. *Brain Research*.

[70] Das UG, Schroeder RE, Hay WW, Devaskar SU (1999). Time-dependent and tissue-specific effects of circulating glucose on fetal ovine glucose transporters. *American Journal of Physiology*.

[71] Thorn SR, Regnault TRH, Brown LD (2009). Intrauterine growth restriction increases fetal hepatic gluconeogenic capacity and reduces messenger ribonucleic acid translation initiation and nutrient sensing in fetal liver and skeletal muscle. *Endocrinology*.

[72] Limesand SW, Rozance PJ, Brown LD, Hay WW (2009). Effects of chronic hypoglycemia and euglycemic correction on lysine metabolism in fetal sheep. *American Journal of Physiology*.

[73] Battaglia FC, Meschia G (1978). Principal substrates of fetal metabolism. *Physiological Reviews*.

[74] Jones CT, Ritchie JWK (1978). The metabolic and endocrine effects of circulating catecholamines in fetal sheep. *Journal of Physiology*.

[75] Barry JS, Davidsen ML, Limesand SW (2006). Developmental changes in ovine myocardial glucose transporters and insulin signaling following hyperthermia-induced intrauterine fetal growth restriction. *Experimental Biology and Medicine*.

[76] Hay WW, DiGiacomo JE, Meznarich HK, Hirst K, Zerbe G (1989). Effects of glucose and insulin on fetal glucose oxidation and oxygen consumption. *American Journal of Physiology*.

[77] Hay WW, Meznarich HK, DiGiacomo JE, Hirst K, Zerbe G (1988). Effects of insulin and glucose concentrations on glucose utilization in fetal sheep. *Pediatric Research*.

[78] Fowden AL, Mijovic J, Silver M (1993). The effects of cortisol on hepatic and renal gluconeogenic enzyme activities in the sheep fetus during late gestation. *Journal of Endocrinology*.

[79] Gentili S, Morrison JL, McMillen IC (2009). Intrauterine growth restriction and differential patterns of hepatic growth and expression of IGF1, PCK2, and HSDL1 mRNA in the sheep fetus in late gestation. *Biology of Reproduction*.

[80] Alexander G, Hales JRS, Stevens D, Donelly JB (1987). Effects of acute and prolonged exposure to heat on regional blood flows in pregnant sheep. *Journal of Developmental Physiology*.

[81] Walker DW, Hale JR, Fawcett AA, Pratt NM (1995). Cardiovascular responses to heat stress in late gestation fetal sheep. *Experimental Physiology*.

[82] Galan HL, Anthony RV, Rigano S (2005). Fetal hypertension and abnormal Doppler velocimetry in an ovine model of intrauterine growth restriction. *American Journal of Obstetrics and Gynecology*.

[83] Regnault TRH, de Vrijer B, Galan HL (2003). The relationship between transplacental O2 diffusion and placental expression of PIGF, VEGF and their receptors in a placental insufficiency model of fetal growth restriction. *Journal of Physiology*.

[84] Wilkening RB, Battaglia FC, Meschia G (1985). The relationship of umbilical glucose uptake to uterine blood flow. *Journal of Developmental Physiology*.

[85] Limesand SW, Jensen J, Hutton JC, Hay WW (2005). Diminished *β*-cell replication contributes to reduced *β*-cell mass in fetal sheep with intrauterine growth restriction. *American Journal of Physiology*.

[86] Greenwood PL, Hunt AS, Hermanson JW, Bell AW (1998). Effects of birth weight and postnatal nutrition on neonatal sheep: I. Body growth and composition, and some aspects of energetic efficiency. *Journal of Animal Science*.

[87] Morrison JL, Duffield JA, Muhlhausler BS, Gentili S, McMillen IC (2010). Fetal growth restriction, catch-up growth and the early origins of insulin resistance and visceral obesity. *Pediatric Nephrology*.

